# Disorder Prediction Methods, Their Applicability to Different Protein Targets and Their Usefulness for Guiding Experimental Studies

**DOI:** 10.3390/ijms160819040

**Published:** 2015-08-13

**Authors:** Jennifer D. Atkins, Samuel Y. Boateng, Thomas Sorensen, Liam J. McGuffin

**Affiliations:** 1 School of Biological Sciences, University of Reading, Whiteknights, Reading RG6 6AS, UK; E-Mails: j.atkins@pgr.reading.ac.uk (J.D.A.); s.boateng@reading.ac.uk (S.Y.B.); 2 Diamond Light Source Ltd., Diamond House, Harwell Science and Innovation Campus, Didcot, Oxfordshire OX11 0DE, UK; E-Mail: thomas.sorensen@diamond.ac.uk

**Keywords:** intrinsic disorder, disorder prediction methods, types of disorder, structural bioinformatics

## Abstract

The role and function of a given protein is dependent on its structure. In recent years, however, numerous studies have highlighted the importance of unstructured, or disordered regions in governing a protein’s function. Disordered proteins have been found to play important roles in pivotal cellular functions, such as DNA binding and signalling cascades. Studying proteins with extended disordered regions is often problematic as they can be challenging to express, purify and crystallise. This means that interpretable experimental data on protein disorder is hard to generate. As a result, predictive computational tools have been developed with the aim of predicting the level and location of disorder within a protein. Currently, over 60 prediction servers exist, utilizing different methods for classifying disorder and different training sets. Here we review several good performing, publicly available prediction methods, comparing their application and discussing how disorder prediction servers can be used to aid the experimental solution of protein structure. The use of disorder prediction methods allows us to adopt a more targeted approach to experimental studies by accurately identifying the boundaries of ordered protein domains so that they may be investigated separately, thereby increasing the likelihood of their successful experimental solution.

## 1. Introduction

**Figure 1:**
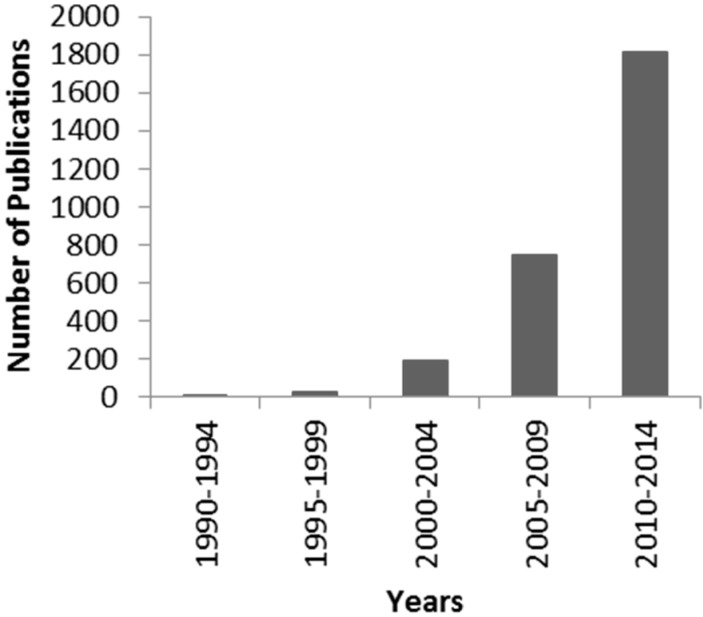
Number of publications relating to intrinsic disorder/unfolded proteins on PubMed since 1990. The early 2000’s saw a dramatic increase in research on these proteins. This figure has been updated from [1] using the same search terms within PubMed; intrinsically disordered, intrinsically unstructured, natively unfolded, intrinsically unfolded and intrinsically flexible.

Intrinsic disorder in proteins has been a hot topic in the molecular sciences since the 1990s. Previously, the function and role of a protein was thought to be characterized by its largely stable and ordered 3D structure. However, it is now known that a high proportion of functionally important regions of proteins contain some level of inherent instability, or intrinsic disorder, and therefore an interest in the study of the phenomenon has risen drastically in the last 20 years (Figure 1) [1]. It has been indicated previously that intrinsically disordered regions of proteins (*i.e.*, regions which do not fold into stable secondary structures) are necessary for performing many functions, such as DNA binding, with at least 28 key functions having been identified [2]. Indeed, approximately a third of all eukaryotic proteins have been identified as including disordered regions greater than 30 residues in length, with 75% of mammalian signalling proteins being somewhat disordered [3].

Disordered regions often cause difficulties for experimental studies of structure, as these regions are inherently flexible, which can make proteins extremely difficult to crystallise, and hence X-ray diffraction analysis may be unfeasible. Experimental data such as those generated via nuclear-magnetic resonance imaging (NMR) or X-ray crystallography (if crystals can be obtained), may be hard to interpret due to random or missing values obtained for the disordered regions [4,5]. Therefore, proteins are often analysed using protein prediction servers prior to experimental analyses to identify disordered regions. If such regions are predicted to exist, perhaps in combination with tertiary structure prediction and molecular dynamics, then mutations and interactions of interest can be modelled to give an idea of how they may affect the protein structure and also to determine which domains may be amenable to further experimental investigation.

## 2. The Importance of Disorder and Disorder Prediction Prior to Experimental Work

Intrinsic disorder is a highly conserved phenomenon and the more “complex” an organism, the greater the levels of disorder that are found within the proteome [3,6,7]. This suggests that disorder may be required for advanced cellular functions and it is therefore of benefit to the organism, possibly because these regions are less sensitive to mutations due to the lack of structure [8]. Various studies, however, have demonstrated that there is a functional element to disorder [1,3,9,10,11,12]. Proteins containing disorder are now understood to be involved in various regulatory roles; intrinsic disorder is believed to allow for binding to multiple targets and also to increase efficiency of binding [13,14]. A key role of disorder is as a flexible linker between two structured domains. The disordered region promotes flexibility of the protein, allowing for the domains to have greater movement, aiding recruitment of binding partners. It could also allow for the protein to have multiple binding partners as binding sites would be open or restricted dependent upon the orientation of the protein in relation to potential binding partners [15]. Ribosomal proteins L7/12, are an example of this; these proteins contain a flexible C-terminal region and are believed to interact with multiple auxiliary translation factors, as well as with the GTPase-associated Region of the ribosome [1]. It has been observed by NMR that L7/12 “tumbles” along the ribosomal body somewhat independently of the ribosome, with the flexible linker region allowing the C-terminus of the proteins to sample various regions of the ribosome [1].

Once bound to a ligand and other subunits, this often promotes a disorder-to-order transition within the protein. An example of this is the case of the nuclear cap-binding protein; in solution, CBP20 alone is fully disordered. However, when CBP20 is part of the nuclear cap-binding complex (CBC), only the N- and C-terminal extensions are disordered [16]. It is therefore thought that binding to the CBP80 subunit induces a structural change. When the CBC is bound to GDP, the CBP20 N- and C-terminal extensions also become ordered [16]. In contrast, a disorder-to-order transition can be created due to a mutation. For example, in the Frizzled 4 (Fz4) cell surface receptor, the cytosolic C-terminal tail of the protein contains disorder however, when the L501fsX533 frameshift mutation is introduced, a helix-loop-helix structure is formed [17]. This mutation is deleterious, which changes the intracellular location of the protein and therefore impedes its activity.

In the case of Fz4, disorder prediction and protein modelling software was utilised to predict the structural change of the receptor which was subsequently confirmed by Circular Dichroism (CD) experiments [17]. Disorder predictions are extremely useful to identify regions of disorder so that manipulations can be made to the protein sequence to aid its expression, purification and crystallisation [18]. A prediction server such as PPC_PRED_, is often used to predict whether a protein is able to be expressed, purified and crystallized. This server in particular incorporates a disorder prediction within the calculations [19]. When used with disorder prediction servers, one can determine areas of disorder which cause issues for one or more of the steps and this may involve either truncating the protein or ensuring that the protein is investigated whilst bound to another protein, or a ligand or metal, in order to induce an ordered state.

In some cases, previously unknown disordered regions have resulted in extended time periods for the resolution of a single protein. The protein NEIL-1 is an example of how disorder prediction can be utilized to target structural studies; originally the authors had attempted crystallisation of the full length sequence [20]. This failed to yield any crystals and so the protein sequence was analyzed using PONDR to investigate any disorder. It was predicted that the C-terminal 106 residues were indeed disordered, however when >100 residues were removed, protein expression was negligible. A construct excluding the C-terminal 100 residues was ultimately chosen and successfully crystallised [20]. This example serves to demonstrate how the initial use of disorder prediction could have potentially saved time as well as costs, leading to a more targeted approach of construct design for crystallisation.

The previous example for truncating a protein is often useful when the disordered region is known to not participate in essential functions, such as substrate binding and glycosylase activity in NEIL-1 [20]. Sometimes however, this is may not be a suitable approach if the disordered region is necessary for function. Depending upon the disorder prediction results, suitable experimental approaches can be adopted. A protein with high levels of disorder (most of the length) would likely prove to be difficult to crystallise even with major sequence edits. In that case, solution based methods, such as CD, NMR or small angle X-ray scattering (SAXS) would be sensible techniques to study the full-length protein chain. CD is a rapid method for the classification of secondary structure of proteins in solution, based upon shifts in optical transitions, with structures defined by wavelength patterns [21]. This method is relatively fast, taking only a few hours for data collection and analysis. NMR on the other hand utilises chemical shifts of individual atoms to identify residues and structure. Disordered proteins can result in overlapping and close standing peaks, making it difficult to determine resonance of a residue [22]. SAXS works by measuring the scatter of X-rays caused by the protein within solution, thereby providing details on the shape and dimensions of the structure [23]. SAXS is often combined with NMR to provide a more thorough analysis, when NMR fails to give an acceptable overall size and shape estimate [24]. However, it is rarely used as the sole method due to its relatively low resolution [25]. In large-scale experimental analyses, the use of predictive tools allows for the exclusion of disordered regions in protein structural determination pipelines, saving time and resources and allowing a focus on ordered regions, for which data are more readily attainable.

## 3. Types of Disorder and Considerations for Predictors

Studying the primary sequence can identify the occurrence of protein disorder. Firstly, such regions often contain fewer hydrophobic amino acids, which prevents the region from forming a hydrophobic core, as is the norm for structured regions [26]. Several studies have investigated the amino acid composition of disordered regions to determine residues that are likely to promote disorder/order [26,27,28]. Across these studies, it is agreed that the residues Serine and Proline are indeed disorder-promoting, however not all studies are in agreement, with each study suggesting several additional disorder promoting residues—Alanine, Arginine, Glycine, Glutamine, Glutamic Acid & Lysine. These studies also investigated order-promoting residues, due to depletion within disordered datasets, with the residues Tryptophan, Cysteine, Phenylalanine, Isoleucine, Tyrosine, Valine, Leucine and Asparagine being considered.

Further to this, disordered regions may have different amino acid compositions (also referred to as different flavours). These differences can have an impact upon the accuracy of a disorder prediction method, depending on which composition or flavour was used as training set for its development. A study undertaken by Vucetic *et al.* revealed that there may be three distinct flavours of disorder based on the composition; these have arbitrarily been named V, C & S [29]. Flavour V contains a greater proportion of less flexible residues Cysteine, Phenylalanine, Isoleucine and Tyrosine than the other flavours, whilst flavour S contains a lower proportion of Histidine residues compared to both ordered regions and the other flavours, and flavour C contains greater proportions of Histidine, Methionine and Alanine than ordered proteins and other flavours [29]. These differences in flavour need to be considered by methods to avoid an over/under prediction of disorder in a given sequence.

Another problematic factor for both predicting and benchmarking disorder predictors is whether the disordered region is considered to be short or long; typically, more than 30 residues is generally classified as a long region of disorder. Dependent upon the composition of the training set, the prediction accuracy for different length regions may be variable. It has been shown in a dataset enriched with short disordered regions (*i.e.*, less than 30 residues) that there is a bias against prediction of long regions of disorder [30]. Previous to this, it had also been shown that predictors trained on long disorder sets resulted in decreased accuracy of short disorder regions due to the sequence composition typical of different length regions [31]. Therefore, when the length of the disordered region is unknown, it is appropriate to utilize either a predictor trained on a mixed data set or a meta-predictor that combines methods trained on different data sets.

## 4. Disorder Prediction Methodologies and Publicly Available Servers

The first disorder prediction method was developed in 1997 [5]. Today, over 60 protein disorder prediction servers exist, although not all are publicly available [32]. These servers are all based upon different methods, with different training sets used in their development. A selection of servers can be found in Table 1. The methods can be classified into four broad categories: Sequence based, clustering, template based and meta-predictor approaches.

*Sequence-based*: The aim of this class of methods is to generate a disorder prediction based purely upon the primary sequence of any given protein. This is done by extracting features from the amino acid sequence itself and/or multiple sequence alignment profiles or scoring matrices in conjunction with statistical models and/or machine learning. This approach was utilized greatly in the CASP8 and CASP9 experiments [33,34,35]. Two methods that use this approach are DISOPRED [36] and PONDR [26]. The DISOPRED server utilizes a method which was trained on 750 non-redundant protein high resolution X-ray crystallography structures [36], assuming disorder for regions where electron density co-ordinates are missing. Although this is a typical method for defining disorder from a known structure, multiple conformations of an ordered domain may also lead to missing electron densities.

PONDR VL-XT also uses missing co-ordinates for classification of the disordered regions found in the eight X-ray structures used for training, however, additionally seven NMR structures with known disorder were used [26]. This predictor combined the VL1 predictor, which was trained on the aforementioned structures with >30 disordered residues, with N- and C-terminal predictors, which were trained on terminal regions of >5 residues. This approach could show accuracy bias favouring long disordered regions, as the terminal short regions may be of a different composition to internal short regions.

**Table 1:** A selection of current protein disorder prediction servers.

Disorder Prediction Server	URL	Description	Publication Date	CASP Rank	Standalone Method Available?
MobiDB [37]	http://mobidb.bio.unipd.it/	10 servers; Espritz (all 3 flavours) [38], IUPred (2 flavours) [39], DisEMBL (2 flavours) [18], GlobPlot [28], VSL2B [31], JRONN [40].	2014	-	No
Metadisorder [32]	http://genesilico.pl/metadisorder/	13 servers; output weighted by accuracy score (S_w_). Uses DisEMBL (3 versions) [18], DISOPRED2 [3], DISpro [41], GlobPlot [28], iPDA [42], IUPred (2 versions) [39], Pdisorder, POODLE-S [43], POODLE-L [44], PrDOS [45], Spritz (2 versions) [46], and RONN [40].	2012	CASP10: 22 CASP9: 14 CASP8: 21	No
Spine-D [47]	http://sparks-lab.org/SPINE-D/	Ab-initio predictor with an initial three-state state prediction. Generates a consensus prediction based upon 5 independent predictors.	2012	CASP9: 4	Yes
MFDp [35]	http://biomine-ws.ece.ualberta.ca/MFDp.html	3 servers; DISOclust [48], DISOPRED [36], IUPred [39].	2010	CASP10: 3/4	No
PreDisorder [49]	http://sysbio.rnet.missouri.edu/predisorder.html	Ab-initio predictor based upon a recursive neural network using a PSI-BLAST profile combined with secondary structure predictions and solvent accessibility.	2009	CASP8: 8	Yes
DISOclust [48]	http://www.reading.ac.uk/bioinf/IntFOLD/	Utilizes outputs from the ModFOLD method to calculate per residue variation in 3D models from IntFOLD.	2008	CASP10: 19 CASP9: 9 CASP8: 3	Yes
metaPrDOS [50]	http://prdos.hgc.jp/cgi-bin/meta/top.cgi	8 servers; prediction scores of each converted into an input vector which feeds into an SVM. Uses PrDos [45], DISOPRED2 [3], DisEMBL [18], DISPROT [31], DISpro [41], IUPred [39], POODLE-S [43], DISOclust [48].	2008	CASP10: 5 CASP8: 13	No
PrDOS [45]	http://prdos.hgc.jp/cgi-bin/top.cgi	Combines two predictors; one based upon amino acid composition and one on template proteins.	2007	CASP10: 1 CASP9: 1	No
POODLE [43,44]	http://mbs.cbrc.jp/poodle/poodle.html	Integrated system using 3 predictors; POODLE-L, POODLE-S and POODLE-W.	2007	CASP10: 6	Yes
DisPro [41]	http://scratch.proteomics.ics.uci.edu/	All disordered X-ray crystal structures from the PDB were filtered to obtain a dataset with only >30 residues. The final data set contained 215, 612 residues; only 6.2% disordered.	2005	-	Yes
IUPred [39]	http://iupred.enzim.hu/	Based upon a quadratic equation of amino acid composition determining energies; chemical type, sequential environment and interaction partners.	2005	-	Yes
DISOPRED 2+3 [36]	http://bioinf.cs.ucl.ac.uk/psipred/	Web based ab-initio prediction server. Trained on 750 non-redundant disordered high resolution X-ray Crystal structures.	2004	CASP10: 2 CASP9: 2 CASP8: 19	Yes
PONDR [26]	http://www.pondr.com/cgi-bin/PONDR/pondr.cgi	Default predictor VL-XT; uses VL1 trained on 8 disordered regions from X-ray crystallographic data and 7 characterized by NMR with >30residues. 10 attributes were used as inputs into a feedforward neural network [26]. This method is combined with the N- and C-terminal predictors to create VL-XT.	1999	-	No

*Clustering:* This approach generates tertiary structure models using the primary sequence and superimposes the models onto each other to identify regions of high variability. The idea is that positions of order should be conserved across multiple models whereas residues that vary are likely to be disordered [33]. An example of this approach is used in DISOclust, which is now integrated with the IntFOLD server [51,52]. The DISOclust method analyses the per residue structural variation across the 3D models generated by the IntFOLD server [48]. As clustering approaches do not rely on the composition of a training set, they may be less likely to show bias regarding disorder length.

*Template-based:* Similar to clustering methods, template based approaches involve aligning the sequence to homologues with known structures. An example of this is PrDOS which utilizes two predictors; one that is amino acid sequence based and another that is template structure based [45]. The theory is that intrinsic disorder should be conserved across protein families. By combining these two approaches, PrDOS could also fall under the meta-predictor category.

*Meta-predictors:* Predictions are made by averaging the outputs of multiple disorder predictors. One example of a meta-predictor is metaPRDOS which combines results from eight different individual methods [50]. Meta predictions often lead to improved accuracy of predictions and are used to populate databases. An example of such a database is the MobiDB, which contains disordered proteins sourced from the PDB and DisProt, exploiting multiple disorder prediction methods [37]. For each protein within the MobiDB, disordered regions are assigned by combining 10 disorder predictors and also by considering the available NMR/X-ray data

**Figure 2:**
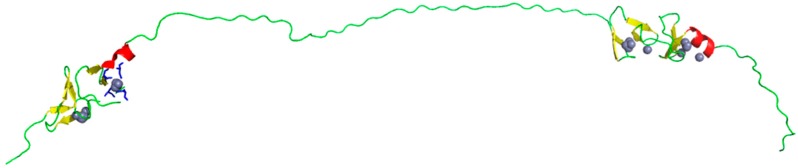
IntFOLD server model of Cardiac MLP. The central and terminal regions are both thought to contain disorder, as found within the other members of the CRP family. The ordered domains are predicted to contain zinc binding sites; likely locations of zinc atoms are indicated by grey spheres. The image is rendered using PyMOL [56].

To demonstrate the difference in disorder predictions between servers, we submitted cardiac Muscle LIM Protein (MLP) (Figure 2) to various servers (Table 2). This protein is known to contain a long disordered region within the central region, similar to other members of the CRP family [53,54]. As with most, if not all, proteins, both the N- and C-termini contain some degree of disorder. The current structures available for this protein however, can be used to investigate the likely true positions of disordered residues; PDB entries 2o10 (residues 7–66) and 2o13 (residues 119–176) resolve only the LIM domains with partial linker sequences included [54]. For the 2o10 construct, residues 1–6 and 72–83 were line broadened but assignable, as were residues 179–187 within the 2o13 construct. Residues 109–112, 136,137,143,156,163 and 183–184 were beyond detection within 2o13 [54]. This suggests that the first seven residues, plus those after residue 66 may contain disorder due to the current structure missing these out. Further, the area between 66 and 119 and from 176–194 likely contains regions of disorder. 

As can be seen in Table 2, it is difficult to identify which prediction server is most correct; all predictors return different results, with some returning vastly different predictions. This example serves to demonstrate the variability of results and reaffirms the need to use multiple servers to get as clear a picture as possible regarding the likelihood of disorder in a given target.

**Table 2:** Comparison of disordered region prediction for Muscle LIM Protein (MLP) from a variety of servers which utilize different methodologies, including the top five ranked servers from the past three CASP (Critical assessment of disorder prediction servers) experiments. N.B. POODLE not tested as unavailable at the time of writing.

Server/Prediction Method	CASP Rank (AUC (ROC) Score)			N-Terminus Disordered Residues	Central Disordered Residues	C-Terminus Disordered Residues
	10	9	8			
PrDOS (5% False Positive; default)	1	1		1–6	-	187–194
PrDOS (15% False Positive)				1	5	184–194
DISOPRED3	2	2	-	1–4	-	185–194
MFDp	3 & 4	7 & 8	19	1–8	87–112	184–194
MFDp2				-	93–108	186–194
metaPrDOS	5	-	13	1–8	82–86, 89–116	187–194
PreDisorder	7	3	8	1–12	41–54, 72–122	151–163, 187–194
Spine-D	9	4	-	1–15	68–119	181–194
DISOclust (From IntFOLD)	19	9	3	1–5	78–123	182–194
GSMetaDisorder	22	14	21	1–5	88–114	182–194
GSMetaDisorderMD	15	10	-	1–5	91–114	185–194
DISOPRED2	-	-	2	1–2	95–114	186–194
GSMetaDisorderMD2	-			1–5	85–115	182–194
MobiDB (consensus)	-			1–6	91–107, 110, 113–118	189–194
PredictProtein: MD	-			1–15	91–119	152, 154–158, 178–194
PredictProtein: UCON	-			49	93–117, 119–121, 126, 133, 136	155–161, 163–164, 187–194
PredictProtein: PROFbval	-			1–16, 18–20, 22, 24, 26–29, 32, 41–48, 50–56	60–131, 136–139	149, 151–165, 170, 173–182, 184–194

Similar server comparisons were carried out by Ferron and colleagues in 2006 [55]. Although disorder predictors have since been improved, this older study also highlighted how variable predictions can be. For example, Heat-Shock Factor binding Protein 1 was known to contain disorder at residues 1–8 and 58–76. RONN and IUPRED were found to predict borderline disorder for the whole protein which is known to be incorrect. As with MLP, the different predictors show varying levels of disorder, for example PreLink predicts 66–76 residues disordered, whereas DISOPRED 2 predicts 1–6, 61–76 and Disembl predicts residues 1–9,62–76 [55]. Based upon this example, DISOPRED2 and Disembl appear to be most reliable methods, with predictions closest to the known disordered regions. These predictors are therefore more accurate for the short regions of disorder than the others of which were tested.

## 5. Critical Assessment of Disorder Prediction Servers

Benchmarking different intrinsic disorder predictors is difficult as they use different approaches; no standard definition of disorder is held across the board and no gold standard method of assignment for disordered regions has been set [29]. This therefore means that different data training sets, containing varying proportions of the three flavours and different distributions of disorder lengths, are utilised dependent upon the author and the methods for self-assessing the accuracy and reliability of the predictions also differ between prediction servers.

The Critical Assessment of protein Structure Prediction (CASP) experiments aim to provide blind objective testing of protein prediction servers to identify current progress and areas of improvement. The CASP experiment has been run every two years since 1994, and has included a protein disorder predictor assessment from 2004 [33]. However, for the 2014 CASP11 experiment, the disorder prediction category was terminated part way through by the assessors due to a lack of “suitable targets” [57].

One of the main issues with the CASP setting is how to compare results from previous experiments to one another. This was first addressed in CASP8, where the assessors compared the S_w_ score (weighted accuracy score of disorder residue prediction) for all targets against that of all minus the protein target that contained a longer disordered region [34]. In doing so, they demonstrated how even slightly different datasets could drastically impact the assessment scores and therefore the CASP forum could be potentially unsuitable for comparison of disorder predictors. For CASP10, the MCC score (Matthew’s correlation coefficient) was held as the main disorder prediction quality score, as it was deemed the most balanced of the three typical binary prediction scores used in CASP9 [58]. When compared to previous CASP experiments, the CASP10 results showed a slight increase in performance. However, as discussed within the paper, this may be biased by the targets used; typically the targets were solved by X-ray crystallography, lending to a bias towards short disorder regions, which would then create a bias in quality score for some predictors [58]. Overall therefore, the results from the CASP experiments, although useful for testing different disorder predictors, cannot be taken at face value due to the problems of finding a sufficient number of suitable targets that would allow for a fair and statistically significant comparison of servers.

A recent study compared a set of 19 disorder prediction programs to assess their suitability for detecting changes in disorder as a result of amino acid substitutions [59]. To do this, Ali *et al.* took experimentally tested examples of substitutions and compared the effects of the residue changes on the disorder predictions [59]. The performance of the prediction servers was based upon the ability to predict the correct disorder/order change of variant residue sites. All servers were deemed to perform poorly as the highest true positive (variants resulting in a structural order change) was >6% and the highest true negative (variants resulting in no order change) correct prediction was 34%. This therefore throws doubt on the ability of disorder prediction servers to detect and correctly predict the changes caused by amino acid substitutions. However, the current servers have not been designed specifically for this purpose and therefore this cannot be held as a test of reliability for disorder prediction. The future direction of disorder prediction therefore could be targeted towards detection of mutational impact. In doing so, studies focused upon the functional impact of mutations would be able to gain a more accurate estimates of the likely structural changes. Despite this, servers which predict the likely effect of mutation currently exist which incorporate a disorder prediction methods in order to make a decision. Examples of this include SIFT-Indel which uses RONN predictions [60] and DDIG-IN using SPINE-D [61]. 

## 6. Conclusions

Currently, no disorder prediction server should be taken in isolation; each has their strengths and weaknesses. In essence, querying a combination of methods and servers, with different attributes for defining disorder, is perhaps the most pragmatic approach to ensure that as true a picture of disorder can be ascertained in the absence of direct experimental evidence. Although we cannot wholeheartedly state that the results obtained are always 100% correct, we can be confident that they do indeed provide us with a highly accurate estimates of the location for disordered regions and therefore give an insight into areas which may prove difficult for experimental structural solution. Furthermore, accurate predictions regarding the location and extent of the intrinsic disorder in proteins allows us to generate new hypotheses about molecular mechanisms and design novel experiments for testing them.
